# Potential Protective Effects of Spirulina (*Spirulina platensis*) against In Vitro Toxicity Induced by Heavy Metals (Cadmium, Mercury, and Lead) on SH-SY5Y Neuroblastoma Cells

**DOI:** 10.3390/ijms242317076

**Published:** 2023-12-03

**Authors:** Rosanna Mallamaci, Maria Maddalena Storelli, Alexia Barbarossa, Giovanni Messina, Anna Valenzano, Daniela Meleleo

**Affiliations:** 1 Department of Biosciences, Biotechnologies and Environment, University of Bari “Aldo Moro”, 70125 Bari, Italy; mariamaddalena.storelli@uniba.it; 2 Department of Pharmacy-Drug Sciences, University of Bari “Aldo Moro”, 70125 Bari, Italy; alexia.barbarossa@uniba.it; 3 Department of Clinical and Experimental Medicine, University of Foggia, 71122 Foggia, Italy; giovanni.messina@unifg.it (G.M.); anna.valenzano@unifg.it (A.V.); 4 Department of Science of Agriculture, Food, Natural Resources and Engineering, University of Foggia, 71122 Foggia, Italy; daniela.meleleo@unifg.it

**Keywords:** *Spirulina*, antioxidants, heavy metals, neurotoxicity, SH-SY5Y

## Abstract

*Spirulina*, a filamentous microalga, is used all over the world as a nutraceutical dietary supplement. Recent studies have focused on examining its chelating activity and antioxidant properties, especially as a candidate for protection against neurotoxicity caused by heavy metals. The MTT test and LDH assay were used to examine the viability of the SH-SY5Y cells for 24, 48, and 72 h, to Cd, Hg, and Pb, individually or in combination with *Spirulina*, and the effects of necrotic cell death. In comparison to the control group, the viability of SH-SY5Y cells decreased after 24 h of exposure, with Cd being more toxic than Hg and Pb being less lethal. The effects of heavy metal toxicity on cell survival were ranked in order after 72 h under identical experimental circumstances as follows: Hg, Pb, and Cd. The viability of the cells was then tested after being exposed to *Spirulina* at doses of 5 at 50 (%*v*/*v*) for 24, 48, and 72 h, respectively. SH-SY5Y cells that had been treated with mixtures of heavy metals and *Spirulina* underwent the same assay. Cell viability is considerably increased by using *Spirulina* treatments at the prescribed periods and doses. Instead, the same procedure, when applied to SH-SY5Y cells, caused the release of LDH, which is consistent with the reduction in cell viability. We demonstrated for the first time, considering all the available data, that *Spirulina* 5, 25, and 50 (%*v*/*v*) enhanced the number of viable SH-SY5Y cells utilized as a model system for brain cells. Overall, the data from the present study provide a first insight into the promising positive role of Spirulina against the potentially toxic effects of metals.

## 1. Introduction

*Spirulina* is a microscopic, unicellular, spiral-shaped alga that is widespread in the brackish and alkaline waters of tropical and subtropical areas. Although falling into the category of blue algae, it is dark green due to the presence of chlorophyll, whose pigments cover the bluish reflections of pyocyanin and the yellow reflections of carotenoids.

*Spirulina* represents a growing special food in the nutrition sector as it is rich in macro- and micronutrients, with high nutritional values: about 60–70% of its dry weight consists of proteins and essential amino acids [1], while the remaining part contains minerals, fatty acids, vitamins, and fat-soluble antioxidants [2]. It also contains polypeptides called phycobiliproteins (e.g., phycocyanin and allophycocyanin) to which the antioxidant activity of the seaweed seems to be linked; moreover, it also appears to have antibacterial activity [3,4].

*Spirulina* promotes anti-inflammatory cytokine secretion, enhances phagocytosis in macrophages, and stimulates immune cells and adaptive humoral immunity [5]; its anti-inflammatory properties may be due to the inhibition of cyclooxygenase-2 (COX-2) [2]. *Spirulina* is made up of polysaccharides that contain many functional groups (e.g., uronic acid and phenolic hydroxyl groups) that participate in the antioxidant and free radical scavenging activities [6] by enhancing the activity of nitric oxide (NO), superoxide dismutase (SOD), catalase (CAT), reduced glutathione (GSH) and glutathione peroxidase (GPX) in liver, kidney, and brain tissues [7].

Several studies have successfully demonstrated that *Spirulina* not only has beneficial effects on the development of the nervous system, but it also has a crucial role in the prevention of oxidative stress [8,9], in the treatment of ROS-induced pathologies, and in the development of new treatments for neurodegenerative diseases such as Alzheimer’s or Parkinson’s [10]. Omid Reza et al., in a randomized, double-blind, controlled trial, demonstrated that the in vivo administration of *Spirulina* is useful for improving brain function and metabolic pathways in Alzheimer’s Disease [11]. Furthermore, it is a rich source of C-Phycocyanin (PCB), an inhibitor of NADPH oxidase that contributes to oxidative stress in various neurological/neurodegenerative diseases [12].

Bermejo-Bescós et al. demonstrated that *Spirulina* prevents iron-induced oxidative stress on SH-SY5Y neuroblastoma cells by increasing the antioxidant enzymes SOD, CAT, GPx, and GR, inhibiting lipid peroxidation activity and reducing high glutathione levels [13,14]. Moreover, *Spirulina* protein extract protects the activity of total GPx, GPx-Se, and GR, and increases reduced GSH through the scavenger activity of free radicals and iron-chelating capacity, as demonstrated through the corresponding enzymatic assays [14]. A growing amount of research shows that using different antioxidants to treat heavy metal toxicity, which results in the production of free radicals in cells, can both prevent and alleviate these negative effects. In reality, numerous studies have demonstrated how antioxidant therapies increase cultured cells’ resistance to dangerous levels of heavy metals [15]. Cadmium (Cd), Mercury (Hg), and Lead (Pb) are heavy metals representing ubiquitous environmental pollutants known to exert serious negative effects on the nervous system, even at low concentrations. On the contrary, metals such as Magnesium (Mg), Manganese (Mn), and Iron (Fe) usually act as enzyme cofactors to set cellular activities but become neurotoxic at high levels. Furthermore, some heavy metals have biological effects, such as antimony and arsenic, with anti-protozoa properties, bismuth for stomach ulcers, gold, which has anti-arthritic effects, and iron, with antimalaria properties [16]. Instead, neurotoxic metals also include the less common metalloid Arsenic (As), which is found in high concentrations in drinking water and food sources in many regions of the world [17]. Disorders such as Alzheimer’s, amyotrophic lateral sclerosis, autism, and Parkinson’s are commonly associated with overexposure to metal. Once metals have accumulated in the nervous system, oxidative stress, mitochondrial dysfunction, and protein misfolding are the most common deficits associated with metal-induced toxicity [18,19,20].

Several preclinical studies demonstrated the alleviative effect of *Spirulina* against experimental Arsenic, Cadmium, Lead, and Mercury toxicities. In addition, some clinical studies have reported protective effects of *Spirulina* against arsenic toxicity in humans [21]. Moreover, it has been reported that *Spirulina* could be a useful tool in the clean-up of polluted water and land areas [22]. A spectroscopic study aimed to assess heavy metals’ absorption by a combined hemp/spirulina system from contaminated soil [23]. With this in mind, in this study, we sought to clarify *Spirulina*’s potential protection against Cd-, Hg-, and Pb-induced neurotoxicity. In this study, we used SH-SY5Y as a model for neuronal cells to emphasize the protective impact of spirulina against Cd, Hg, and Pb at three different exposure times.

## 2. Results

### 2.1. Total Phenolic Content and Antioxidant Activity

The total phenolic content (TPC) of *Spirulina* was determined with the Folin–Ciocalteau method and the results obtained are expressed as g/L gallic acid equivalent (GAE). Total phenolic content amounted to 0.2599 + 0.02 g/L GAE. The antioxidant capacity was evaluated using the oxygen radical scavenging capacity (ORAC) assay, for which a value is equal to 23,887 + 329.43 µmolTE/g. High, positive and significant correlations were found between antioxidant capacity (ORAC) and total polyphenols. This suggests that the presence of polyphenols could contribute to antioxidant activity.

### 2.2. Effect of Cd, Hg, Pb or Spirulina on Cell

To determine the individual effects of Cd, Hg, and Pb on the human SH-SY5Y neuronal cells, we performed cytotoxicity experiments, treating the cells with increased concentrations of each metal ion for different incubation times. The results of the MTT show that the cytotoxic effect induced by two heavy metals in neuroblastoma cells is dependent on the metal concentration and exposure time. This assay is used to measure cell viability and is based on the reduction of MTT to a blue formazan product by dehydrogenase enzymes in intact mitochondria. Thus, the MTT assay measures mitochondrial function or integrity.

Cd increased cytotoxicity in a time- and concentration-dependent manner. At doses of 0.25, 2.5, 25, and 250 µM Cd, the survival rate drastically dropped over 24 h, going from 86, 75, and 54 to 29%, respectively. Following 48 and 72 h of exposure to 25 µM Cd, the survival rate fell to 42 and 35%, respectively; following 48 and 72 h of exposure to 250 µM Cd, all cells were nearly dead in comparison to the control.

Hg was less toxic to SH-SY5Y cells compared to Cd: at 24 h, the survival rate decreased to 78 and 34% at 25 and 250 μM Hg, respectively; after 48 h, the survival rate decreased to 63 and 31% at 25 μM and 250 μM Hg, respectively; after 72 h, it decreased to 62 and 25% at 25 μM and 250 μM Hg, respectively, compared to control.

Also, Pb cytotoxicity was lower than cadmium and constant in the concentration range of 0.25 and 2.5 μm at all established times of treatment. On 24 h, the survival rate decreased to 87 and 65% at 25 and 250 μM Pb, respectively; after 48 h, the survival rate decreased to 77 and 67% at 25 μM and 250 μM Pb, respectively; after 72 h, it decreased to 77 and 54% at 25 μM and 250 μM Pb, respectively, compared to control. When lead is exposed to high levels, we notice that the effect is time-dependent.

According to the results of the first experimental set, a 24 h exposure period is adequate to comprehend the negative effects of the three metal ions, which become drastically greater with a longer exposure (72 h). These results support the following toxicity scale for SH-SY5Y cells: before Hg and Pb is Cd, which demonstrated the highest toxicity. Additionally, the obtained results served as a basis for the choice of the concentration of heavy metals, which have a significantly decreased vitality (25 μM) in the evaluation of the potential protective action of the tested substance.

**Figure 1:**
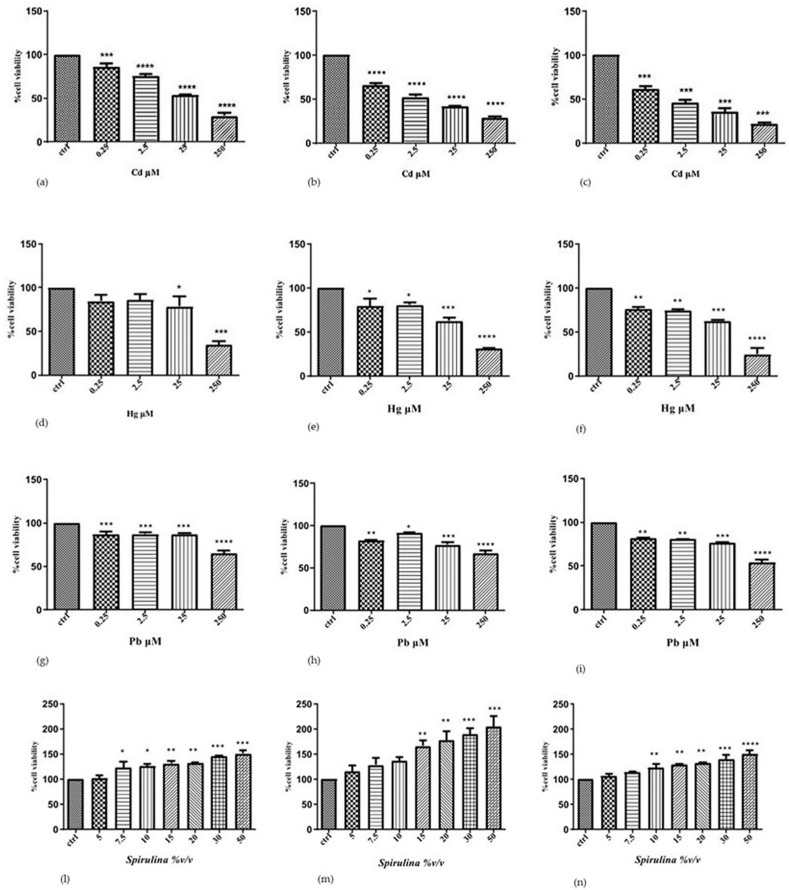
Effect of CdCl_2,_ HgCl_2,_ PbCl_2,_ and *Spirulina* on SH-SY5Y cell viability after 24 h (**a**,**d**,**g**,**l**), 48 h (**b**,**e**,**h**,**m**) and 72 h (**c**,**f**,**i**,**n**) of exposure (MTT assay). Data are expressed as a percentage of vehicle-treated cells (control). Results are shown as mean ± SEM (*n* = 3). * *p* < 0.05, ** *p* < 0.01, *** *p* < 0.001, **** *p* < 0.0001 compared with the control. The different patterns in the histograms show varying concentrations of heavy-metals and *Spirulina*.

To assess *Spirulina*’s effectiveness, SH-SY5Y cells were additionally exposed to concentrations of 5, 7.5, 10, 15, 20, 30, and 50 (%*v*/*v*) for 24, 48, and 72 h, respectively. The results revealed a significant dose-dependent increase in cell viability compared to the control after 24 and 48 h. The dose–time-dependent effect, however, becomes less pronounced after 72 h of exposure (Figure 1).

### 2.3. Effect of Spirulina on Cd-, Hg-, and Pb-Induced Cytotoxicity

**Figure 2:**
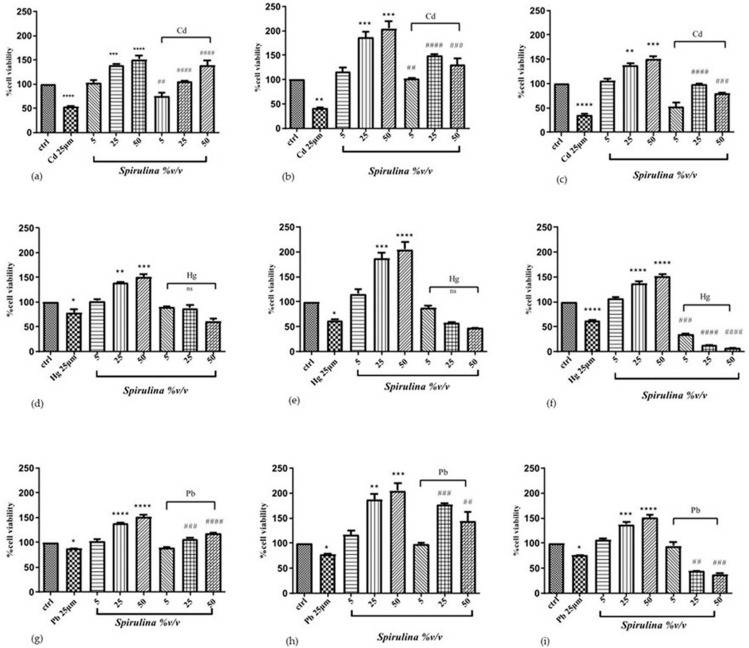
Effect of simultaneous treatment with CdCl_2_, HgCl_2_, PbCl_2,_ and *Spirulina* on SH-SY5Y cell viability after 24 h (**a**,**d**,**g**), 48 h (**b**,**e**,**h**) and 72 h (**c**,**f**,**i**) of exposure (MTT assay). Data are expressed as mean ± SEM (*n* = 3). Significant differences versus control * *p* < 0.05, ** *p* < 0.01, *** *p* < 0.001, **** *p* < 0.0001. ^##^ *p* < 0.01, ^###^*p* < 0.001, ^####^ *p* < 0.0001 as compared to CdCl_2_, HgCl_2_, PbCl_2_ alone. The different patterns in the histograms show varying concentrations of heavy-metals and *Spirulina*.

To determine whether *Spirulina* can protect cells from Cd, Hg, and Pb toxicity, three different exposure times were used, 24, 48, and 72 h, with the following concentrations: *Spirulina* 5, 25, and 50% *v*/*v* and Cd, Hg, and Pb 25 μM, respectively. Cells were simultaneously treated with *Spirulina* at different concentrations; for the heavy metals, the 25 μM concentration was chosen because it significantly reduced cell viability but did not cause total cell death [24]. Cell viability, evaluated using the MTT assay, was significantly reduced for cells exposed to each heavy metal compared with control cells. This effect was significantly ameliorated when cells were treated with *Spirulina*. The results showed that *Spirulina* has a time- and concentration-dependent cytoprotective effect against nearly all three heavy metals. Simultaneous treatment with Cd or Pb and *Spirulina* showed an increase in cell viability up to 21, 52, and 84%, and up to 2, 20, and 17% after 24 h of treatment with *Spirulina* used at concentrations of 5, 25, and 50 (%*v*/*v*), respectively, compared to the cells treated with Cd or Pb only. A remarkable cytoprotective effect was observed after 48 h treatment with Cd and Pb/*Spirulina* mixtures, increasing cell vitality up to 61, 108, and 89%, and up to 20, 100, and 67%, compared with the Cd- or Pb-treated cells. However, the 72 h treatment was not very advantageous, probably due to the excessive exposure of the cells to the Pb. The cytoprotective effect is only exerted against Cd, with an increase in cell vitality of 18, 64, and 45% of the mixture compared with the Cd-treated cells. Regarding the effects of SH-SY5Y cells’ exposure to mixtures of Hg/*Spirulina,* an increase in cell viability of up to 12 and 25% was observed after 24 h and 48 h of exposure with *Spirulina* 5% *v/v,* and an increase of only up to 10% was observed after 24 h with *Spirulina* 25% *v/v* compared with the Hg-treated cells. After 48 and 72 h exposure, at the other remaining mixture concentrations, an increase in cell viability was not observed. In SH-SY5Y cells, heavy metals impaired MTT reduction, which was partially recuperated in the presence of *Spirulina.* These findings suggest that mitochondrial defense mechanisms play a role in the protection of SH-SY5Y cells with *Spirulina.* The effect of Cd, Hg, and Pb with or without the presence of *Spirulina* is shown in Figure 2.

### 2.4. The Effect of Spirulina on Cd-, Hg-, and Pb-Induced LDH Release

To examine the probability of oxidative-stress-induced membrane damage, we assessed the potential protective effect of *Spirulina* on heavy-metals-induced cytotoxicity using the LDH assay, measuring the activity of this stable enzyme when released into the medium regarding apoptosis, necrosis, and other events of cellular injury in SH-SY5Y cells. Membrane integrity declines during necrosis or late apoptosis, and LDH is released into the environment. It is possible to quantify LDH activity and estimate the proportion of cells that have died through cell lysis. Treatment with Cd and Hg alone significantly elevated LDH release by up to 61% and 44%, respectively, in SH-SY5Y after 48 h. However, for lead alone, there was no discernible rise in LDH release at the time of treatment. In contrast, LDH release was dramatically reduced when *Spirulina* 5, 25, and 50 (%*v*/*v*) were administered.

**Figure 3:**
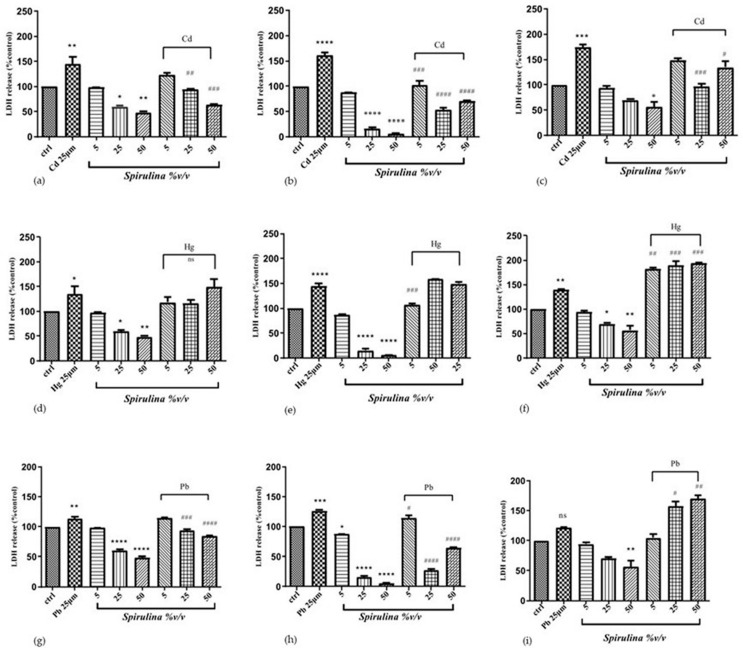
LDH activity of SH-SY5Y cell after 24 h (**a**,**d**,**g**), 48 h (**b**,**e**,**h**) and 72 h (**c**,**f**,**i**) treatment with different concentrations of *Spirulina* and CdCl_2_, HgCl_2,_ and PbCl_2_. Data are expressed as mean ± SEM (*n* = 3). Significant differences versus control * *p* < 0.05, ** *p* < 0.01, *** *p* < 0.001, **** *p* < 0.0001. ^#^ *p* < 0.05, ^##^ *p* < 0.01, ^###^ *p* < 0.001, ^####^ *p* < 0.0001 as compared to CdCl_2_, HgCl_2_, PbCl_2_ alone. The different patterns in the histograms show varying concentrations of heavy-metals and *Spirulina*.

When SH-SY5Y cells were exposed to mixtures of Cd/*Spirulina,* LDH release significantly decreased up to 58%, 110%, and 91% after 48 h of treatment compared to Cd-treated cells, and 20% and 28% after Pb/*Spirulina* treatment at 25 and 50 (%*v*/*v*), compared to Pb-treated cells after only 24 h. Regarding Hg, no reduction in LDH release was seen at any dose or time during concomitant *Spirulina* treatment (Figure 3).

## 3. Discussion

A significant global health issue is environmental exposure to neurotoxic metals, including cadmium, mercury, and lead [25]. Depending on the duration of exposure, they may contribute to the etiology of neurodegenerative illnesses like Alzheimer’s (AD) and Parkinson’s (PD), which affect the brain’s ability to function in old age. Reactive oxygen species (ROS) interactions with sulfhydryl chemical groups (-SH) in proteins, and their competition for essential metal binding sites (such as Fe, Cu, and Zn), are all part of the molecular activities studied when looking at the toxicity of heavy metals in various biological models [26]. Additionally, cellular apoptosis, necrosis, and other cellular damage events cause a significant increase in LDH release in response to cadmium, mercury, and lead toxicity [27]. The importance of good nutrition in reducing heavy-metals-induced toxicity is frequently emphasized. Numerous authors hypothesized that different antioxidants could both prevent and treat the harmful effects of heavy metals, which result in the body’s production of free radicals. The toxicity of this metal can be reduced by other foods, even if some meals can enhance human exposure [28]. Currently, naturally occurring substances that can chelate heavy metals are used to increase gastric-intestinal transit to aid in heavy metal elimination [29]. One of these commercially available “superfoods”, with thousands of distinct biomolecules (phytochemicals), some of which can enhance health and/or prevent disease, is the subject of the current investigation. The most frequently grown microalga in the world is *Spirulina,* which is also the most well-known genus among microalgae and consists of the cyanobacteria species *Arthrospira platensis* and *Arthrospira maxima*. *Spirulina* is well known for having a high protein content, but it also contains significant amounts of iron, magnesium, potassium, carotenoids, phycobiliproteins, B vitamins, and many other vitamins, minerals, and antioxidants [30]. However, several health advantages have been identified, including immune system enhancement, protection against cardiovascular and chronic degenerative diseases, and antioxidant and detoxifying properties [31] *Spirulina* protects PC12 cells from the toxicity induced by iron. After *Spirulina* treatment, a linear rise in antioxidant enzymes, such as CAT, peroxidase (PX), SOD, and ascorbate peroxidase (APx), was seen. Additionally, *Spirulina* increases the levels of hydrophilic antioxidants (glutathione and ascorbic acid) and lipophilic antioxidants (total carotenoids and α-tocopherol) in cells, reversing the damage caused by increasing H_2_O_2_ concentrations.

The current work used the MTT assay, a widely used test for cell viability, to demonstrate the cytoprotective abilities of *Spirulina* on SH-SY5Y neuroblastoma cells against heavy metals (cadmium, mercury, and lead). First, the findings indicate that, under the conditions of our experiment, heavy metals cause cytotoxicity that is dose- and time-dependent, with Cd being more toxic than Hg and Pb being less lethal [32]. This difference could be due to the different mechanisms of heavy metal uptake by SH-SY5Y cells. Previous research has shown that Cd exposure (0–40 µM) reduces cell viability in a dose-dependent manner and disrupts the cytoskeleton in cultured neuroblastoma cells, affecting signal transmission and cell function and causing neurotoxicity [33]. In a study on optic nerves exposed to 200 µM Cd for 100 min, identical outcomes of axonal damage, defined by cytoskeleton disruption as well as mitochondrial disruption, were seen [34]. This suggests a similar mechanism for Cd-induced white matter injury [35] There has been extensive research on MeHg-treated SH-SY5Y cells. After a 24 h incubation period, Sanfeliu, C et al. (2001) found that the lethal concentration (LC_50_) value for MeHg was 6.9 µM [36]. Methylmercury was administered to SH-SY5Y cells by Toimela and Tähti (2004) [32] at concentrations ranging from 0.01 to 1000 µM, with 1 µM serving as the effective concentration (EC) for 24 h. It was discovered that methylmercury damages SH-SY5Y cells in a dose- and time-dependent manner. Following exposure to MeHg, MMP activity decreased while caspase 3 activity increased after exposure to MeHg at doses of 0.1, 1, and 10 µM after 6, 24, and 48 h [37]. When the SH-SY5Y cell line was exposed to Pb (from 0.01 to 10 µM), it significantly and concentration-dependently reduced the growth of neuroblastoma cells. Around 5 µM Pb caused a 50% (IC50) inhibition of cell proliferation. The effects of Pb exposure include changes to the Golgi apparatus, dysfunctional mitochondria, an increase in glial filaments in astrocytes, and oxidative stress. Lead further reduces glutathione levels by inactivating glutathione-S-transferase [38].

The findings of our investigation show that each of the three metals under examination causes a decrease in cell viability and an increase in the rate of cell death when added to cultivated SH-SY5Y neuroblastoma cells.

Next, with the addition of *Spirulina* to the intact cells, the viability of the cells increased and a concentration- and time-dependent cytoprotective effect was seen. Our assessment of the antioxidant potential of the phenols in *Spirulina* implies that it is a source of antioxidants and can be used for both food and non-food applications. The ability of *Spirulina* to increase cell viability and lower cell death rate in a culture could be related to its direct antioxidant potential. Phycocyanobilin, a phytochemical abundant in *Spirulina,* is thought to limit NADPH oxidase activity, enhance glutathione synthesis, and produce a notable amount of antioxidant enzymes, all of which may be used to manage oxidative stress in cell disease [39,40]. It is well recognized that oxidative stress can be a component of the harmful effect of heavy metals. We showed, for the first time, that *Spirulina* 5, 25, and 50 (%*v*/*v*) could prevent cell death caused by Cd, Hg, and Pb using SH-SY5Y cells as a model system for brain cells. While *Spirulina* has a strong protective impact against Cd at all treatment times, it has a protective effect against Hg at 24 h and against Pb at both 24 and 48 h. According to a previous theory, the various processes used by SH-SH5Y cells to absorb heavy metals account for their varied sensitivity to metals. Our findings also demonstrate that metal sensitivity is maintained across various treatments, indicating that the type of metal always determines the concentration that a cell can absorb.

One of the most common types of physiologically recognized cell death is necrosis. We assessed LDH leakage, a general marker of cell membrane damage and necrotic cell death, to investigate the impact of neurotoxic substances on cell death [41] Significant LDH leakage was seen in cells treated with CdCl_2_ or PbCl_2_ at concentrations that resulted in a considerable decline in cell viability, showing that the induction of necrosis was one of the main drivers of the reduction in cell viability [42]. In our study using *Spirulina,* we found that the treatment of SH-SY5Y cells with the heavy metals from the outer scales and *Spirulina* led to an increase in cell viability and a decrease in enzyme release, which were indicators of the cell’s resistance to the heavy metals. After 24 h of treatment, cells incubated with Cd and Pb/*Spirulina mixes* significantly lessened the negative effects caused by 25 µM heavy metal alone compared to cells treated with *Spirulina* 50% (*v*/*v*). After receiving Cd and Pb/*Spirulina* mixes for 48 h, considerable cytoprotective activity was observed, with a notable effect at the initial concentration of *Spirulina* at 25% (*v*/*v*) compared to heavy metals alone. Due to the limited protective impact of *Spirulina,* which is consistent with the results regarding vitality, there was likely no reduction in LDH release in the Hg/*Spirulina* group.

Our research revealed that SH-SY5Y cells proliferated more after being incubated with *Spirulina* at concentrations of 5, 25, and 50 (%*v*/*v*) for 24–48–72 h. This suggests that the obtained filtrate may be able to activate the proteins vital to cell survival. As shown by the treatment of neuronal cells with both *Spirulina* and a heavy metal solution, the proliferative effect of our *Spirulina* platensis on SH-SY5Y cells may be attributed to its neuroprotective action against heavy-metals-induced cell damage [43]. Our observations lead us to the conclusion that Cd-, Hg-, and Pb-induced oxidative stress causes cell death, most likely through necrosis. However, the treatment of cells with phycobiliprotein-rich *Spirulina* inhibits LDH release and shields SH-SY5Y cells from neurotoxicity. According to the degree of the potential protective effect, i.e., lowering the cell death rate, the studied heavy metals can be placed in the following sequence: Cd > Pb > Hg.

In addition to the antioxidant capacity, the decrease in the cell death rate after the administration of *Spirulina* together with heavy metals may be due to the synergistic effects of all its nutritional components, as well as a hypothetical ability of *Spirulina* to form complexes with metal ions [44]. According to Tsukihara et al., 1978, the chelating ability of *Spirulina* derives from a chloroplast-type ferredoxin in the active center whose efficiency appears to be influenced by many physical and chemical factors, such as initial metal concentration, dosage, time of adsorption, temperature, and pH [45].

## 4. Materials and Methods

### 4.1. Chemicals and Reagents

*Arthrospira platensis*: strain was cultivated by ApuliaKundi S.r.l. in open ponds (3000 L per pound) under controlled greenhouse conditions with natural light at a temperature ranging from 22 to 28 °C. The production cycle was monitored by turbidity measurements through a Secchi disk (200 mm diameter, Scubla, Remanzacco (UD), Italy). Once the disk was lowered into the algae suspension at 5÷6 cm and could not be seen anymore, the algae were collected. Overall, the production cycle lasted approximately 3 days. The collected algal biomass was filtered (40 μm filter), extruded, and cold-dried at a temperature value lower than 38 °C. The study was conducted using dried biomass in powder form, commonly named *Spirulina.* CdCl_2_, HgCl_2_ or PbCl_2,_ high-glucose (4.5 gL^−1^) Dulbecco’s modified Eagle medium (DMEM), fetal bovine serum (FBS, PAN Biotech, Aidenbach, Germany), L-glutamine, trypsin, MTT, sodium pyruvate, potassium phosphate, NADH, were purchased from Sigma-Aldrich S.p.a. (Milan, Italy).

### 4.2. Determination of Total Phenolic Content

The total phenolic content of *Spirulina* was determined using the Folin–Ciocalteu method with some modification [46]. In brief, 100 µL of compound solution (1 mgmL^−1^ MeOH) was shaken for 1 min with 1 mL of diluted (1:10) Folin–Ciocalteu reagent. Then 800 µL of 10% Na_2_CO_3_ was added, and the final volume was 5.0 mL with distilled water. After the mixture was left to stand for 2 h at room temperature, the absorbance at 760 nm was measured using a UV–vis spectrophotometer (model V-550, Jasco, Europe). The results of total phenolic content were estimated using a standard curve prepared using gallic acid and expressed as g/L gallic acid equivalents (GAE).

### 4.3. Preparation of Spirulina and Heavy Metals Solutions

One day before the experiments, *Spirulina* powder (10 mg) was soaked in complete culture medium (1 mL) with the 2% addition of antibiotic–antimycotic solution. The mixture was shaken continuously and incubated overnight at 37 °C. The obtained supernatant was collected and filtrated using syringe filters, first with a 0.45 µm pore size, then using filters with a 0.22 µm pore size. The obtained filtrate was maintained at 4 °C [47]. Cadmium, Mercury, and Lead were administered as the water-soluble salt CdCl_2_, HgCl_2,_ or PbCl_2_. A stock solution of CdCl_2_, HgCl_2,_ or PbCl_2_ was prepared by dissolving under stirring 0.2283 and 0.2715 g, and 0.2811 g, respectively, of CdCl_2_, HgCl_2,_ or PbCl_2_ powder in bidistilled sterile water (10 mL) and subsequent filtration. The final concentration was 1 × 10^−1^ M. The CdCl_2_, HgCl_2,_ or PbCl_2_ solutions at concentrations ranging from 0.01 to 250 μM were obtained by scalar dilution of the stock solution. All the solutions were stored at 4 °C until use.

### 4.4. Antioxidant Capacity

The oxygen radical absorbance capacity (ORAC) method was performed to determine the antioxidant capacity of the filtrate by monitoring the inhibition of the action of free peroxyl radicals formed by the decomposition of 2,2-azobis (2-methylpropionamide)- dihydrochloride (AAPH) against the fluorescent compound fluorescein [46]. In brief, 150 µL of fluorescein and 25 µL of the sample in 1:200 dilution (or Trolox in the case of the standard compound, or puffer in the case of blank) were pipetted into microplate wells and thermostated for 30 min at 37 °C. After 30 min, 25 µL of AAPH was added and measurements were performed at excitation and emission wavelengths of 485 and 520 nm every minute for 80 min. The results were expressed as µM of Trolox Equivalents (µM TE). The EC_50_ value was determined by a dose–response curve with at least 6 concentrations.

### 4.5. Culture Cells

Human SH-SY5Y neuroblastoma cells were used in this study ((ATCC^®^ CRL2266™) were purchased from American Type Culture Collection (ATCC, Manasass, VA, USA)). The cells were normally cultured in 25 cm_2_ flasks (T-25 flask, Corning, New York, USA) and maintained in high glucose (4.5 gL_−1_) Dulbecco’s modified Eagle medium (DMEM), supplemented with 10% (*v*/*v*) fetal bovine serum (FBS, PAN Biotech, Aidenbach, Germany), 4 mM L-glutamine (Sigma-Aldrich (St. Louis, MO, USA), and 1% (*v*/*v*) antibiotic solution penicillin-streptomycin (Lonza Bioscience; Walkersville, MD, USA). The cells were maintained at 37 °C in an incubator (Thermo Scientific Hera Cell 240i, Waltham, MA, USA) with 5% CO_2_ in the air atmosphere and 95% relative humidity. The complete growth medium (DMEM) was changed every two days. After becoming 80% confluent, the cells were washed with phosphate-buffered saline solution (PBS) to remove any unattached cells. The attached cells were harvested using a 1 mL 0.25% trypsin and 0.53 mM EDTA solution (SIGMA) and then seeded at a density of 5 × 10^3^ cells/well in a 96-well plate and incubated for 24 h to allow for attachment.

To monitor the toxicity of CdCl_2_, HgCl_2,_ or PbCl_2_ on SH-SY5Y neuroblastoma cells, we prepared the following experimental sets:

In the first experimental set, cells were exposed to increasing concentrations of tree heavy metals (0.25, 2.5, 25, and 250 μM) CdCl_2_ HgCl_2_, or PbCl_2_ in 6 wells per concentration group, and incubated for 24, 48, and 72 h. Heavy metal was added to the culture media individually.

In the second set of experiments, cells were exposed to increasing concentrations of *Spirulina* filtrate 5, 7.5, 10, 15, 20, 30, and 50 (%*v*/*v*), and incubated for 24, 48, and 72 h.

In the third experimental set, cells were treated with mixtures of CdCl_2_ HgCl_2_, or PbCl_2_ (25 μM)/filtrate at scalar concentrations of 5, 25, and 50 (%*v*/*v*), and incubated for 24, 48, and 72 h. Control groups consisted of SH-SY5Y cells, which were processed in the same manner and incubated simultaneously with the treated groups [48].

### 4.6. Determination of Cell Viability

The MTT (3-(4,5-dimethylthiazol-2-yl)-2,5- diphenyltetrazolium bromide) technique, which relies on the ability of mitochondrial oxidoreductases to convert soluble MTT into insoluble formazan in live cells, was used to determine the vitality of the cells. The amount of formazan that is generated corresponds to the number of live cells [49] In brief, SH-SY5Y cells were seeded at a density of 5 × 10^4^ in a 96-well plate (6 wells/concentration group *plus* 1 control group) and, following various treatments, cells were incubated with 20 μL of MTT stock solution (5 mg/mL in PBS 1X) in 180 μL of medium in the dark. After an additional three hours at 37 °C, the medium was taken out, 150 μL of DMSO was added to dissolve the formazan crystals, and the mixture was kept warm for five minutes while being stirred. The absorbance was recorded at 540 nm with a multilabel microplate reader (Wallac Victor3, 1420 Multilabel Counter, Perkin-Elmer (Waltham, MA, USA). All MTT assays were performed in triplicate. Cell viability is expressed as a percentage of the control group (% control) calculated from the equation: *% control* =*Absorbance treatment/Absorbance control* × 100%. Data are the mean percentages of viable cells vs. the respective controls.

### 4.7. Lactate Dehydrogenase (LDH) Release

Lactate dehydrogenase (LDH) leakage into the culture medium was used to measure cytotoxicity. To obtain a cell-free supernatant, the media were sucked and centrifuged for 5 min. at 3000 rpm after being exposed to the CdCl_2_, HgCl_2,_ or PbCl_2_ and Spirulina. The supernatant was used for assay of LDH activity. In brief, the reaction was initiated by mixing 0.1 mL of cell-free medium with 48 mM potassium phosphate buffer (pH 7.5) containing 0.18 mM NADH and 0.6 mM sodium pyruvate in a final volume of 3 mL. A microplate spectrophotometer system (Bio-Rad 680; Bio-Rad Laboratories, Inc.) was used to measure the change in absorbance at 440 nm. The formula for calculating cell LDH release is as follows: (% control) is: % control = (U LDH/mg cell protein) treatment/(U LDH/mg cell protein) control 100% [50,51].

### 4.8. Statistical Analysis

Data are shown as the mean ±SEM. Multiple comparisons were evaluated with one-way analysis of variance (ANOVA), followed by Bonferroni or Dunnett’s test, using the statistical package in the GraphPad Prism software vers.9.01 (GraphPad Software, Inc., San Diego, CA, USA) [52,53]. Differences were considered significant at *p* < 0.05.

## 5. Conclusions

The outcomes of this investigation reveal that exposure to heavy metals damages SH-SY5Y cells in a dose- and time-dependent way.

The *Spirulina* investigated in this research has good protective efficacy against SH-SY5Y cells exposed to CdCl_2_, HgCl_2_, and PbCl_2_, although in varying amounts. Indeed, cell viability markedly increased in cells that were co-treated with heavy metals and *Spirulina* compared to cells exposed to heavy metals alone.

Additional evidence that the multiple advantageous substances present in the microalga, including carotenes, antioxidants, and pigments like chlorophyll and phycocyanin, might affect cells comes from the toxicity detected through the measurement of LDH. These effects are reversed by therapy with *Spirulina*.

In the final section, our study demonstrates how the increased resistance of cultured cells to heavy metal toxicity may be an indication of the potential in vivo protective role of *Spirulina*, especially for neurological tissues, and should be an inspiration for further research aiming to achieving practical relevance [11].

Although the presented data are interesting, it is essential to conduct additional in vitro experiments using various neuronal cellular models to validate these findings and explore the possible neuroprotective mechanisms of *Spirulina*. This will provide a better understanding of the benefits of *Spirulina* and its potential role in preventing or treating neurological disorders.

## Data Availability

The datasets generated during the current study are available from the corresponding author upon reasonable request.

## References

[1] Soni R.A., Sudhakar K., Rana R.S. (2017). *Spirulina—*From growth to nutritional product: A review. Trends Food Sci. Technol..

[2] Reddy C.M., Bhat V.B., Kiranmai G., Reddy M.N., Reddanna P., Madyastha K.M. (2000). Selective Inhibition of Cyclooxygenase-2 by C-Phycocyanin, Biliprotein from Spirulina platensis. Biochem. Biophys. Res. Commun..

[3] Abdel-Moneim A.-M.E., El-Saadony M.T., Shehata A.M., Saad A.M., Aldhumri S.A., Ouda S.M., Mesalam N.M. (2021). Antioxidant and antimicrobial activities of *Spirulina platensis* extracts and biogenic selenium nanoparticles against selected pathogenic bacteria and fungi. Saudi J. Biol. Sci..

[4] Kurd F., Samavati V. (2015). Water soluble polysaccharides from *Spirulina platensis*: Extraction and in vitro anti-cancer activity. Int. J. Biol. Macromol..

[5] Maddaly R. (2010). The beneficial effects of spirulina focusing on its immunomodulatory and antioxidant properties. Nutr. Diet. Suppl..

[6] Han P., Li J., Zhong H., Xie J., Zhang P., Lu Q., Li J., Xu P., Chen P., Leng L. (2021). Anti-oxidation properties and therapeutic potentials of spirulina. Algal Res..

[7] Thaakur S.R., Jyothi B. (2007). Effect of spirulina maxima on the haloperidol induced tardive dyskinesia and oxidative stress in rats. J. Neural Transm..

[8] Sorrenti V., Castagna D.A., Fortinguerra S., Buriani A., Scapagnini G., Willcox D.C. (2021). Spirulina Microalgae and Brain Health: A Scoping Review of Experimental and Clinical Evidence. Mar. Drugs.

[9] Caetano P.A., do Nascimento T.C., Fernandes A.S., Nass P.P., Vieira K.R., Maróstica J.M.R., Jacob-Lopes E., Zepka L.Q. (2022). Microalgae-based polysaccharides: Insights on production, applications, analysis, and future challenges. Biocatal. Agric. Biotechnol..

[10] Kiziltan H.S. (2021). Handbook of Oxidative Stress in Cancer: Therapeutic Aspects.

[11] Omid Reza T., Heidari-Soureshjani R., Asemi Z., Kouchaki E. (2023). The effects of spirulina intake on clinical and metabolic parameters in Alzheimer’s disease: A randomized, double-blind, controlled trial. Phytother. Res..

[12] Trotta T., Porro C., Cianciulli A., Panaro M.A. (2022). Beneficial Effects of Spirulina Consumption on Brain Health. Nutrients.

[13] Maqoud F., Scala R., Tragni V., Pierri C.L., Perrone M.G., Scilimati A., Tricarico D. (2021). Zoledronic Acid as a Novel Dual Blocker of KIR6.1/2-SUR2 Subunits of ATP-Sensitive K^+^ Channels: Role in the Adverse Drug Reactions. Pharmaceutics.

[14] Bermejo-Bescós P., Piñero-Estrada E., del Fresno Á.M.V. (2008). Neuroprotection by *Spirulina platensis* protean extract and phycocyanin against iron-induced toxicity in SH-SY5Y neuroblastoma cells. Toxicol. In Vitro.

[15] Kumar A., Ramamoorthy D., Verma D.K., Kumar A., Kumar N., Kanak K.R., Marwein B.M., Mohan K. (2022). Antioxidant and phytonutrient activities of *Spirulina platensis*. Energy Nexus.

[16] Gemma C., Mesches M.H., Sepesi B., Choo K., Holmes D.B., Bickford P.C. (2002). Diets enriched in foods with high antioxidant activity reverse age-induced decreases in cerebellar beta-adrenergic function and increases in proinflammatory cytokines. J. Neurosci..

[17] Lima E., Guerra R., Lara V., Guzmán A. (2013). Gold nanoparticles as efficient antimicrobial agents for *Escherichia coli* and Salmonella typhi. Chem. Cent. J..

[18] Gade M., Comfort N., Re D.B. (2021). Sex-specific neurotoxic effects of heavy metal pollutants: Epidemiological, experimental evidence, and candidate mechanisms metals neurotoxicity sex-specific neurotoxic effects heavy metals sexual dimorphism brain. Environ. Res..

[19] Meleleo D., Sblano C., Storelli M.M., Mallamaci R. (2020). Evidence of cadmium and mercury involvement in the Aβ42 aggregation process. Biophys. Chem..

[20] Meleleo D., Gerbino A., Mastrodonato M. (2022). Evidence of the different effect of mercury and cadmium on the hIAPP aggregation process. Biophys. Chem..

[21] Bhattacharya S. (2020). The role of Spirulina (Arthrospira) in the mitigation of heavy-metal toxicity: An appraisal. J. Environ. Pathol. Toxicol. Oncol..

[22] Murali O., Mehar S.K. (2014). Bioremediation of heavy metals using Spirulina. Int. J. Geol. Earth Environ. Sci..

[23] Musio B., Ahmed E.M.F.M.H., Antonicelli M., Chiapperini D., Dursi O., Grieco F., Latronico M., Mastrorilli P., Ragone R., Settanni R. (2022). A spectroscopic study to assess heavy metals absorption by a combined hemp/spirulina system from contaminated soil. Environ. Adv..

[24] Marrelli M., Argentieri M.P., Alexa E., Meleleo D., Statti G., Avato P., Conforti F., Mallamaci R. (2022). Antioxidant activity and protective effect of the outer scales hydroalcoholic extract of Allium cepa L. var. Tropea on toxicity damage induced by Cadmium in Caco-2 cells. Food Chem. Toxicol..

[25] Tchounwou P.B., Yedjou C.G., Patlolla A.K., Sutton D.J. (2012). Heavy Metal Toxicity and the Environment. Mol. Clin. Environ. Toxicol..

[26] Carmona A., Roudeau S., Ortega R. (2021). Molecular Mechanisms of Environmental Metal Neurotoxicity: A Focus on the Interactions of Metals with Synapse Structure and Function. Toxics.

[27] Genchi G., Sinicropi M.S., Lauria G., Carocci A., Catalano A. (2020). The Effects of Cadmium Toxicity. Int. J. Environ. Res. Public Health.

[28] Jaishankar M., Tseten T., Anbalagan N., Mathew B.B., Beeregowda K.N. (2014). Toxicity, mechanism and health effects of some heavy metals. Interdiscip. Toxicol..

[29] Notariale R., Infantino R., Palazzo E., Manna C. (2021). Erythrocytes as a Model for Heavy Metal-Related Vascular Dysfunction: The Protective Effect of Dietary Components. Int. J. Mol. Sci..

[30] Jung F., Krüger-Genge A., Waldeck P., Küpper J.-H. (2019). *Spirulina platensis*, a super food?. J. Cell Biotechnol..

[31] Rehman M.U., Wali A.F., Ahmad A., Shakeel S., Rasool S., Ali R., Rashid S.M., Madkhali H., Ganaie M.A., Khan R. (2019). Neuroprotective Strategies for Neurological Disorders by Natural Products: An update. Curr. Neuropharmacol..

[32] Toimela T., Tähti H. (2004). Mitochondrial viability and apoptosis induced by aluminum, mercuric mercury and methylmercury in cell lines of neural origin. Arch. Toxicol..

[33] Chen L., Liu L., Huang S. (2008). Cadmium activates the mitogen-activated protein kinase (MAPK) pathway via induction of reactive oxygen species and inhibition of protein phosphatases 2A and 5. Free Radic. Biol. Med..

[34] Xie Z., Zhang Y., Li A., Li P., Ji W., Huang D. (2010). Cd-induced apoptosis was mediated by the release of Ca2+ from intracellular Ca storage. Toxicol. Lett..

[35] Fern R., Black J.A., Ransom B.R., Waxman S.G. (1996). Cd(2+)-induced injury in CNS white matter. J. Neurophysiol..

[36] Sanfeliu C., Sebastià J., Kim S.U. (2001). Methylmercury Neurotoxicity in Cultures of Human Neurons, Astrocytes, Neuroblastoma Cells. Neurotoxicology.

[37] Toimela T., Mäenpää H., Mannerström M., Tähti H. (2004). Development of an in vitro blood–brain barrier model—Cytotoxicity of mercury and aluminum. Toxicol. Appl. Pharmacol..

[38] Suresh C., Dennis A.O., Heinz J., Vemuri M.C., Chetty C.S. (2006). Melatonin Protection against Lead-Induced Changes in Human Neuroblastoma Cell Cultures. Int. J. Toxicol..

[39] Li Y. (2022). The Bioactivities of Phycocyanobilin from Spirulina. J. Immunol. Res..

[40] Stanic-Vucinic D., Minic S., Nikolic M.R., Velickovic T.C. (2018). Spirulina Phycobiliproteins as Food Components and Complements. Microalgal Biotechnology.

[41] Méry B., Guy J.-B., Vallard A., Espenel S., Ardail D., Rodriguez-Lafrasse C., Rancoule C., Magné N. (2017). In Vitro Cell Death Determination for Drug Discovery: A Landscape Review of Real Issues. J. Cell Death.

[42] Ortega D.R., Esquivel D.F.G., Ayala T.B., Pineda B., Manzo S.G., Quino J.M., Mora P.C., de la Cruz V.P. (2021). Cognitive Impairment Induced by Lead Exposure during Lifespan: Mechanisms of Lead Neurotoxicity. Toxics.

[43] Li Y., Shi W., Li Y., Zhou Y., Hu X., Song C., Ma H., Wang C., Li Y. (2008). Neuroprotective effects of chlorogenic acid against apoptosis of PC12 cells induced by methylmercury. Environ. Toxicol. Pharmacol..

[44] Machu L., Misurcova L., Vavra Ambrozova J., Orsavova J., Mlcek J., Sochor J., Jurikova T. (2015). Phenolic Content and Antioxidant Capacity in Algal Food Products. Molecules.

[45] Tsukihara T., Fukuyama K., Tahara H., Katsube Y., Matsuura Y., Tanaka N., Kakudo M., Wada K., Matsubara H. (1978). X-Ray Analysis of Ferredoxin from *Spirulina platensis*: II. Chelate Structure of Active Center. J. Biochem..

[46] Dávalos A., Gómez-Cordovés C., Bartolomé B. (2004). Extending Applicability of the Oxygen Radical Absorbance Capacity (ORAC−Fluorescein) Assay. J. Agric. Food Chem..

[47] Śmieszek A., Giezek E., Chrapiec M., Murat M., Mucha A., Michalak I., Marycz K. (2017). The Influence of *Spirulina platensis* Filtrates on Caco-2 Proliferative Activity and Expression of Apoptosis-Related microRNAs and mRNA. Mar. Drugs.

[48] Romero A., Ramos E., Castellano V., Martínez M.A., Ares I., Martínez M., Martínez-Larrañaga M.R., Anadón A. (2012). Cytotoxicity induced by deltamethrin and its metabolites in SH-SY5Y cells can be differentially prevented by selected antioxidants. Toxicol. In Vitro.

[49] Amoroso S., Gioielli A., Cataldi M., Di Renzo G., Annunziato L. (1999). In the neuronal cell line SH-SY5Y, oxidative stress-induced free radical overproduction causes cell death without any participation of intracellular Ca^2+^ increase. Biochim. Biophys. Acta (BBA) Mol. Cell Res..

[50] Kaja S., Payne A.J., Naumchuk Y., Koulen P. (2017). Quantification of Lactate Dehydrogenase for Cell Viability Testing Using Cell Lines and Primary Cultured Astrocytes. Curr. Protoc. Toxicol..

[51] Sun A.Y., Chen Y.-M., James-Kracke M., Wixom P., Cheng Y. (1997). Ethanol-Induced Cell Death by Lipid Peroxidation in PC12 Cells. Neurochem. Res..

[52] Scala R., Maqoud F., Antonacci M., Dibenedetto J.R., Perrone M.G., Scilimati A., Castillo K., Latorre R., Conte D., Bendahhou S. (2022). Bisphosphonates targeting ion channels and musculoskeletal effects. Front. Pharmacol..

[53] Maqoud F., Vacca E., Tommaseo-Ponzetta M. (2016). From Morocco to Italy: How Women’s Bodies Reflect their Change of Residence. Coll. Antropol..

